# Keep Calm and Comp. Cog. On. Commentary: A crisis in comparative psychology: where have all the undergraduates gone?

**DOI:** 10.3389/fpsyg.2016.00020

**Published:** 2016-01-25

**Authors:** Valerie A. Kuhlmeier, Mary C. Olmstead

**Affiliations:** Department of Psychology, Queen's UniversityKingston, ON, Canada

**Keywords:** comparative psychology, comparative cognition, undergraduates, graduate programs, education, cognition, evolution

Full Disclosure: We have recently authored an undergraduate textbook entitled *Comparative Cognition* (Olmstead and Kuhlmeier, 2015). Our motivation to write this book did not result from a perceived lack of undergraduate interest in the topic, but rather *overenrolled* courses for which we did not have a textbook.

A key difference between our approach—largely shared by fellow commenters Brodbeck and Brodbeck (2015) and McMillan and Sturdy (2015)—and that of Abramson (2015), is our willingness to work under the title “comparative cognition.” We have been trained under various titles (e.g., biological anthropology, behavioral ecology, ethology, cognitive psychology, developmental psychology, and behavioral neuroscience) and have witnessed the continued emergence of comparative cognition as the logical intersection of these disciplines. Though, still considered a narrowly focused field by Abramson, we propose instead that comparative cognition has broadened (or perhaps always was broad, see Hulse et al., 1978; Wasserman, 1981), and encompasses, without conflict, Abramson's defining features of comparative psychology.

Our point here goes beyond an argument about semantics, though it is important to be clear about our use of “comparative cognition,” Take, for example, a statement in the commentary by Bielert and Gallup (2015): “In particular, comparative cognition is quite similar to comparative psychology aside from restricting its research questions and measures to those specific to information processing (Shettleworth, 2010).” We do not see this “restriction” in scope in the modern practice of comparative cognition research. Instead, researchers consider the ways that general learning mechanisms may be constrained by early developing (perhaps innate) behavioral or perceptual biases, conceptual abilities, and/or non-conceptual processes.

So yes, comparative cognition is open to the possibility of cognitive processes, but it actively tests for them. It also goes far beyond comparing behavioral responses of “species X” to that of humans (an anthropocentric approach that only a subset of researchers adopt, often with appropriate consideration of phylogenetics), and gone are the days of studying only a few, unrelated species. To use one metric: at the 2015 meeting of the Comparative Cognition Society, research with over 20 different species was presented. Shettleworth (2009), a pioneer and leader in the field, noted that comparative cognition research in the first decade of the twenty-first century was characterized by an increasing number of species examined and more synergy with related fields, such as behavioral ecology.

A scholar of comparative cognition—and we think Shettleworth would agree with this based on her 2010 book—should be knowledgeable about sensory systems, behavioral tenets including general associative learning mechanisms, memory, and proposed cognitive mechanisms that are implicated in such areas as categorization, spatial navigation, number, timing, prosocial behavior, communication, and social learning, all the while considering an evolutionary, developmental, and neuroscientific framework. It's a tall order, but what an exciting field!

In our experience, undergraduates agree. Like fellow commenters Furlong et al. (2015), we see the “missing” undergraduates every week—200 of them this term alone in a second year survey course: *Introduction to Comparative Cognition*. Many of these students will take our upper level laboratory courses that focus on specialized topics within the field of comparative cognition. The majority of these students specialize in other areas of psychology such as developmental, clinical, or cognitive psychology, a number are biology or life science majors, and a handful come from complementary disciplines such as computer science or philosophy. And yes, occasionally students seek out graduate study in laboratories specifically focused on comparative cognition. Regardless, we trust that they all pursue their chosen area of study with a greater appreciation and engagement in the basic, comparative science that informs so many fields of study.

## Author contributions

All authors listed, have made substantial, direct and intellectual contribution to the work, and approved it for publication.

## Funding

This work was funded by a Discovery Grant to VK from the Natural Sciences and Engineering Council of Canada.

### Conflict of interest statement

The authors declare that the research was conducted in the absence of any commercial or financial relationships that could be construed as a potential conflict of interest.
